# DAILY FLUCTUATIONS IN SUBJECTIVE AGE AND MENTAL HEALTH: THE ROLES OF CHRONOLOGICAL AGE AND ATTITUDES TO AGING

**DOI:** 10.1093/geroni/igz038.200

**Published:** 2019-11-08

**Authors:** Dikla Segel-Karpas, Amit Shrira, Ehud Bodner

**Affiliations:** 1 University of Haifa, Haifa, Israel, Israel; 2 Bar-Ilan University, Ramat Gan, HaMerkaz, Israel

## Abstract

Studies indicate that subjective age – individuals’ perception of their own age as older or younger than their chronological age, is related to their depressive symptoms. Less is known about the role that attitudes towards aging might play in this regard. 334 participants (age 30-90, M=58.15) reported their subjective age and depressive symptoms every day for a period of 14 days. Attitudes to aging were measured at baseline. Results indicated that daily subjective age was related to daily variation in depressive symptoms. Furthermore, we found that attitudes to aging (psychosocial losses, gains and physical changes) moderated the subjective age-depression relationship, such that it was stronger when psychosocial losses were high, and when physical changes and gains were low. The moderating effect of losses was especially prominent for older participants. This indicates that the general perception of aging moderates the toll that feeling old takes on mental health.

